# BMP2 peptide-modified polycaprolactone-collagen nanosheets for periodontal tissue regeneration

**DOI:** 10.3389/fbioe.2025.1523735

**Published:** 2025-03-05

**Authors:** Yong Zhang, Junxian Ren, Zongshan Shen, Jiayu Yang, Jichen Yang, Zhengmei Lin, Xuetao Shi, Chuanjiang Zhao, Juan Xia

**Affiliations:** ^1^ Guangdong Provincial Key Laboratory of Stomatology, Guanghua School of Stomatology, Hospital of Stomatology, Sun Yat-sen University, Guangzhou, Guangdong, China; ^2^ National Engineering Research Centre for Tissue Restoration and Reconstruction, South China University of Technology, Guangzhou, Guangdong, China; ^3^ Key Laboratory of Biomedical Engineering of Guangdong Province, South China University of Technology, Guangzhou, Guangdong, China

**Keywords:** BMP2 peptide, polycaprolactone-collagen nanosheets, barrier membranes, periodontal tissue regeneration, RNA sequencing

## Abstract

**Introduction:**

Periodontitis leads to the degradation of tooth-supporting tissues, ultimately causing tooth mobility and loss. Guided tissue regeneration (GTR) surgery employs barrier membranes to facilitate tissue regeneration. However, conventional membranes lack bone-inducing properties, thereby limiting their efficacy. Our objective was to develop a bifunctional GTR membrane that combines mechanical stability with bone-inducing capabilities. To achieve this, we engineered BMP2 peptide-modified polycaprolactone-collagen nanosheets (BPCNs) to enhance periodontal regeneration by improving cell adhesion, osteogenesis, and anti-inflammatory activity.

**Methods:**

BPCNs with nanoscale thickness were fabricated using the spin-coating technique, incorporating BMP2 peptides, collagen, polycaprolactone (PCL), and polyvinyl alcohol (PVA). Successful conjugation of BMP2 to the BPCNs was verified through UV spectrophotometry and confocal laser scanning microscopy. The biocompatibility and cell adhesion properties of BPCNs were rigorously assessed using CCK-8 assays, microscopic imaging, and quantitative cell counting. *In vitro* osteogenic efficacy was evaluated by Alizarin Red S (ARS) staining and quantitative reverse transcription polymerase chain reaction (qRT-PCR) to analyze osteogenic marker gene expression. A rat periodontal defect model was established to assess *in vivo* regenerative performance, with outcomes analyzed through micro-CT, hematoxylin-eosin (H&E) staining, and Masson’s trichrome staining, confirming enhanced tissue regeneration and the absence of systemic toxicity. The mechanistic pathways underlying BPCNs-mediated regeneration were elucidated via RNA sequencing (RNA-seq), revealing the activation of osteogenic signaling cascades and the suppression of proinflammatory pathways.

**Results:**

BPCNs demonstrated excellent biocompatibility, promoted fibroblast and bone marrow stem cell (BMSC) adhesion, and enhanced BMSC osteogenesis. Furthermore, BPCNs significantly promoted periodontal tissue regeneration in a rat model. Mechanistically, RNA-seq analysis revealed that BPCNs upregulated genes involved in tissue regeneration and downregulated proinflammatory pathways.

**Discussion:**

This study introduced a novel osteoinductive nanosheet, termed BPCNs, which provides a groundbreaking material-based approach for the regenerative repair of periodontal tissue defects. These findings position BPCNs as a highly promising candidate for GTR surgery, with significant potential to improve clinical outcomes in periodontal regenerative medicine.

## 1 Introduction

Periodontitis, one of the most common chronic inflammatory diseases in humans, is a chronic infectious disease caused by pathogenic microorganisms. It is characterized by the progressive and irreversible destruction of tooth-supporting tissues, which eventually leads to tooth loss (Papapanou et al., 2018), affecting patient quality of life (Shen Z. et al., 2024). Moreover, periodontitis is closely related to a series of systemic diseases, such as diabetes (Sanz et al., 2018), inflammatory bowel disease (IBD) (Wang et al., 2023), and Alzheimer’s disease (Shen Z. S. et al., 2024). The existing treatment approaches of periodontal scaling and root planing have been proven to effectively control mild and moderate periodontitis (Suvan et al., 2020), but restoring and regenerating damaged alveolar bone remains challenging (Huang et al., 2024).

Guided tissue regeneration (GTR) technology provides temporal and spatial support for both soft tissue and hard tissue repair by covering the barrier membrane in the bone defect area to block the growth of soft tissue, which helps to promote bone tissue and periodontal ligament regrowth and therefore periodontal regeneration (Donos et al., 2023; Francisco et al., 2019; Sanz et al., 2019). However, traditional GTR membranes are limited in clinical applications because they lack bone induction ability and are difficult to use (Dwivedi et al., 2020), which hinders their effectiveness in promoting bone regeneration. Building on recent advances in nanotechnology, researchers have recently begun applying nanotechnology in the context of tissue regeneration, and the developed materials been named nanosheets (Shi et al., 2014; Hamed et al., 2023). Based on our previous research, our nanosheets materials exhibit several notable advantages over existing membrane materials, including thinner dimensions, ease of manipulation, superior wet adhesion performance, and excellent mechanical strength. Recently, our group has further demonstrated that nanosheets, with a thickness of tens of nanometers, exhibit not only biocompatibility, biodegradability, and unique physical properties such as high adhesive ability and flexibility, but also an exceptional ability to adapt to the moist oral environment, thereby rendering them highly suitable for oral clinical applications (Fujie et al., 2009; Fu et al., 2023). However, the potential of nanosheets for periodontal tissue regeneration remains unclear.

Nanosheets are typically combined with collagen layers to load bioactive peptides for specific applications, such as promoting bone regeneration. Among the factors studied, the BMP-2 peptide is recognized for its ability to stimulate osteoblast differentiation, proliferation, and adhesion, thereby enhancing bone regeneration (Kim et al., 2013). Combination of the BMP-2 peptide with nanosheets not only supports the formation of bone tissue but also has the potential to induce bone regeneration. Therefore, the synergistic effect of the BMP-2 peptide and nanosheets provides a new idea for the development of a new generation of GTR membranes.

**FIGURE 1:**
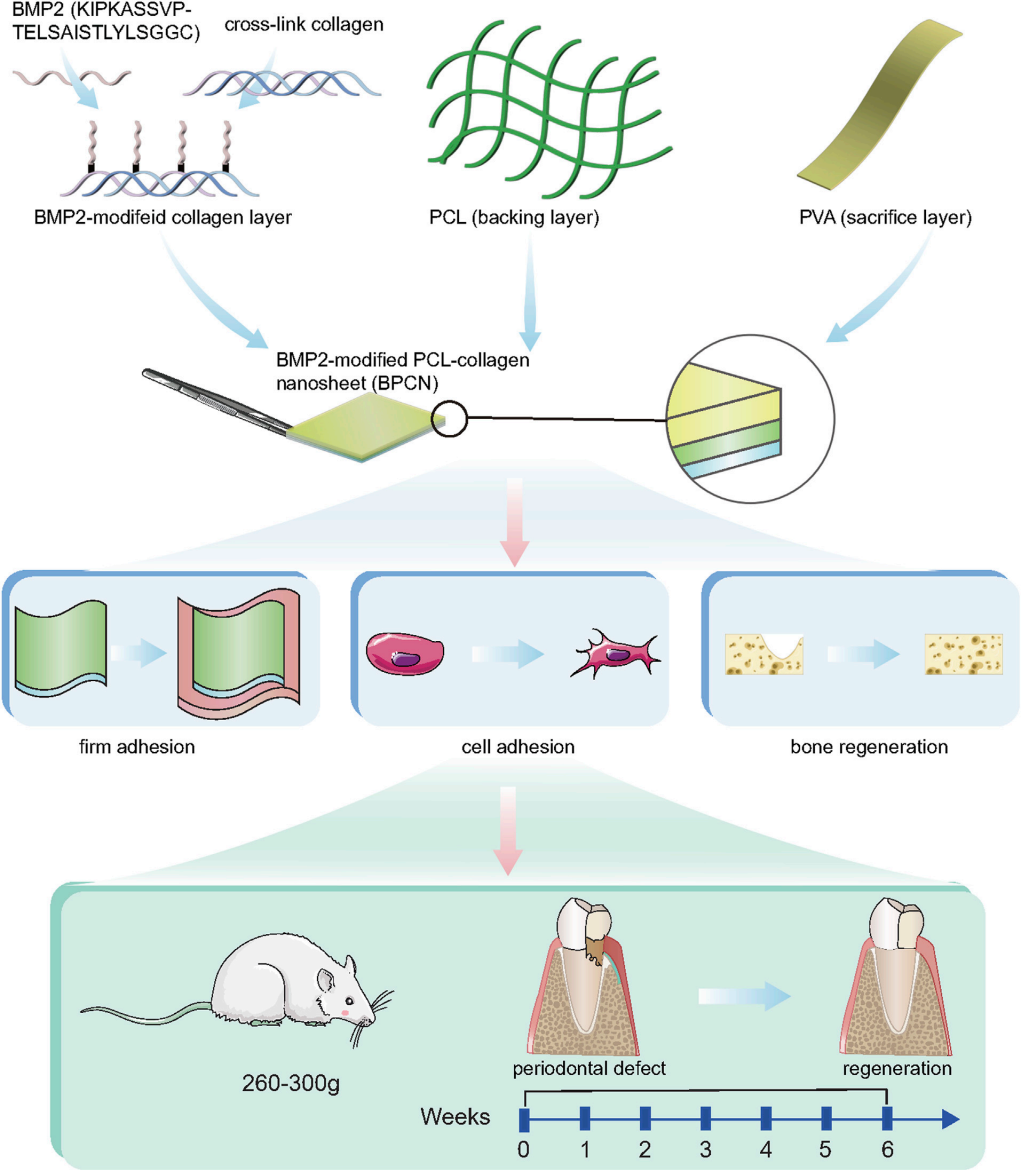
Components and properties of BPCNs and their application in GTR.

In this context, we developed BMP2 peptide-modified PCL-collagen nanosheets (BPCNs) for GTR. These multifunctional nanosheets have good adhesion and mechanical properties and are easy to use when combined with bone-inducing growth factors. Therefore, this innovative method is expected to overcome the current limitations of GTR by providing a bionic and bone-inducing platform and promoting alveolar bone regeneration. We evaluated the osteogenic ability and biocompatibility of the BPCNs through a series of experiments. In addition, we constructed a rat alveolar bone defect model and evaluated the therapeutic effect of BPCNs during GTR (Figure 1). Overall, in comparison to existing commercial guided tissue regeneration (GTR) membranes, our research has culminated in the development of multifunctional biodegradable polymeric composite nanofilms (BPCNs) utilizing a spin-coating technique, which facilitates rapid and large-scale production. When benchmarked against previously studied GTR membranes, our BPCNs exhibit several notable advantages, including a thinner profile, ease of manipulation, superior wet adhesion properties, and enhanced mechanical strength. Furthermore, our BPCNs demonstrate superior adaptability to the moist oral environment. Notably, when loaded with bone morphogenetic protein-2 (BMP2), our BPCNs exhibit osteogenic induction capabilities. Our study demonstrates that these multifunctional nanosheets may be promising GTR membrane materials for the treatment of alveolar bone defects.

## 2 Materials and methods

### 2.1 Reagents used to prepare BPCNs

Polyvinyl alcohol (PVA, Mw: ∼13,000), hexafluoroisopropanol, polycaprolactone (PCL, Mw: ∼80,000), 1-ethyl-3-(3-dimethylaminopropyl) carbodiimide (EDC), N-hydroxy succinimide (NHS) and ethylenediaminetetraacetic acid (EDTA) were purchased from Aladdin (Shanghai, China). Collagen type I was purchased from Beijing Allgens Medical Science and Technology Co. Ltd. 4-(N-Maleimidomethyl) cyclohexane-1-carboxylic acid 3-sulfo-N-hydroxysuccinimide ester sodium salt (Sulfo-SMCC) was purchased from Beijing Biosynthesis Biotechnology Co. Ltd. KIPKASSVPTELSAISTLYLSGGC and FITC-labeled KIPKASSVPTELSAISTLYLSGGC were obtained from ChinaPeptides Co., Ltd. (Shanghai, China).

### 2.2 Preparation process for BPCNs

The preparation process for BPCNs can be referenced in our previous studies. Briefly, the thickness of the nanosheets is directly proportional to the concentrations of both the PCL and collagen solutions (Xuan et al., 2020). Previous research demonstrate that a 0.75 wt% collagen solution and a 10 mg/mL PCL solution are suitable for the preparation of BPCNs. Type I collagen was dissolved in a hexafluoroisopropanol solution and stirred overnight to obtain a 0.75 wt% collagen solution. Subsequently, EDC and NHS were mixed with the collagen solution at a mass ratio of collagen I:EDC:NHS of 6:1:1.

The collagen solution was then dripped onto a silicon wafer and spun at 4,000–6,000 rpm min^−1^ for 30 s to form collagen nanosheets (CNs). After rinsing with pure water, the collagen layer was soaked in Sulfo-SMCC buffer solution (1 mg/mL) for 1 h and then incubated with KIPKASSVPTELSAISTLYLSGGC (purity >95%) EDTA solution (0.5 mg/mL) at 4°C overnight to produce BMP2-modified collagen nanosheets (BCNs). Additionally, a PCL solution of 15 mg/mL was prepared in dichloromethane. This solution was then dripped onto the BCNs, spun at 4,000–6,000 rpm min^−1^ for 30 s, and allowed to dry, resulting in BMP2 peptide-modified PCL-collagen nanosheets (BPCNs). After the BPCNs were rinsed several times with pure water, a 20 wt% PVA solution was added dropwise, and the mixture was spun at 2000–3,000 rpm min^−1^ for 30 s. Owing to the presence of PVA as a sacrificial layer, the nanosheets could be easily peeled off using tweezers after drying.

### 2.3 Characterization of BPCNs

To determine the grafted amount of KIPKASSVPTELSAISTLYLSGGC, we measured the concentration difference via Ellman’s method. Initially, KIPKASSVPTELSAISTLYLSGGC was dissolved in PBS to produce a 0.5 mg/mL BMP-2 peptide solution. This solution was subsequently combined with Ellman’s reagent (supplied by MedChemExpress, United States), and the absorbance at 412 nm was subsequently determined using a microplate reader (Bio Tek Epoch, United States). After a standard curve for the OD value was established, the change in the concentration of the peptide following grafting was calculated. Additionally, KIPKASSVPTELSAISTLYLSGGC that was labeled with fluorescein isothiocyanate (FITC) and had a purity of over 95% was utilized to prepare BPCNs. Grafting of BMP-2 onto the nanosheets was then observed via confocal laser scanning microscopy (Zeiss LSM980, Germany).

Fifty microliters of deionized water was applied to both the BPCNs and BCNs for 1 hour, allowing us to observe the hydrophilicity and water resistance properties of the PCL and collagen layers. After removing the PVA sacrificial layer, the surface morphology of the BPCNs and BCNs was analyzed via field emission scanning electron microscopy (SEM; Zeiss, Germany). Additionally, the cross-sectional and surface morphologies of the BPCNs were examined via atomic force microscopy (AFM; Zeiss, Germany), and the thickness of the BPCNs was accurately measured.

The mechanical properties of the nanosheets were tested at room temperature with a universal tensile testing machine (CMT4204, SANS). The nanosheets were cut into a dumbbell shape. Later, the samples were fixed onto the device and subjected to a consistent tensile force until the nanosheets fractured to evaluate the tensile strength and generate the stress‒strain curves. The Young’s moduli were calculated from the slope of the initial linear region of the stress‒strain curve.

### 2.4 Cellular behavior, biocompatibility and cell adhesion testing of BPCNs

L929 cells and bone marrow stem cells (BMSCs) were used to assess the biocompatibility of the BPCNs and their ability to promote L929 cell and BMSC adhesion. The experimental groups consisted of L929 cells and BMSCs cultured on both the collagen layer of PCL-collagen nanosheets (PCNs) and the BMP2-modified collagen layer of BPCNs. The reference substrates for comparisons of cell adhesion were twenty-four-well plates (Wuxi NEST Biotechnology Co., Ltd.) made of tissue culture-treated polystyrene (TCPS), which exhibited excellent hydrophilicity and cell adhesion (Lerman et al., 2018).

After being subjected to ultraviolet sterilization for 60 min, PCNs and BPCNs were securely attached to 24-well plates. L929 cells and BMSCs were subsequently inoculated into each well at a density of 5 × 10^4^ cells per well. After the cell suspension was washed away with PBS at 2 h and 4 h post-inoculation, microscopic observation was conducted and images were captured. The numbers of adherent cells were counted in six random visual fields. To assess cell proliferation on days 1, 2, and 3, CCK-8 reagent (Sigma‒Aldrich, United States) was used, and the OD value was determined using a microplate reader (Bio Tek Epoch, United States).

### 2.5 *In vitro* osteogenic properties of BPCNs

BMSCs were seeded into six-well plates in osteogenic medium (Cyagen, China) at a density of 5 × 10³ cells per well. They were then cultured with PCNs, BMP2 peptides and BPCNs for 21 days. Following 21 days of coculture, the cells were fixed with a 4% paraformaldehyde solution, washed three times with PBS, and subsequently stained with ARS staining kits (Cyagen, China). Quantification was performed using ImageJ software. Additionally, after 14 days of coculture, cells from different treatment groups were collected to assess the expression of osteogenic genes, including osteopontin (OPN), osteocalcin (OCN), and alkaline phosphatase (ALP), through quantitative real-time polymerase chain reaction (qRT‒PCR) assays. The primers used are listed in Supplementary Table S1.

### 2.6 *In vivo* assessments

Twenty male SD rats (weights: 260–300 g) were purchased from Sun Yat-sen University. All experiments were approved by the Animal Care and Use Committee of Sun Yat-sen University under protocol number SYSU-IACUC-2021-001051. The SD rats were randomly assigned to four groups, with five animals in each group. The groups were as follows: (1) the PBS group, which included rats with periodontal defects but no treatment; (2) the PCN-treated group; (3) the BMP2 peptide-treated group; and (4) the BPCN-treated group. To evaluate the biological performance of the nanosheets *in vivo*, we used a rat periodontal defect model. The animals were anesthetized using pentobarbital sodium (supplied by MREDA Technology; lot number 1507002). A small incision was made on the gingiva before the first left maxillary molar, revealing the alveolar bone under continuous saline irrigation. A periodontal defect, approximately 1 mm × 1 mm × 1 mm, was created on the mesial alveolar bone of the first maxillary molar using a small electric drill. The membranes were subsequently placed over the defects, and the incisions were sutured. Six weeks after surgery, the animals were euthanized, and the maxillary bones with defects were collected.

### 2.7 Micro-CT analysis

For micro-CT analysis, the maxillary bones of the experimental rats were collected, fixed in 4% PFA for 24 h, washed three times with PBS, dehydrated in 75% ethanol, placed in standardized cylindrical sample containers, and then scanned using a high-resolution micro-CT scanner (Scano Medical AG, Bassersdorf, Switzerland). The parameters were set to 70 kV, 114 mA, 20 μm increments, and a 3,000 ms integration time. Following scanning, three-dimensional microstructural image data were reconstructed and analyzed using image analysis software (Mimics Research 21.0, Materialize, Belgium). The distance between the cementoenamel junction and the alveolar bone crest (CEJ-ABC distance) was measured.

### 2.8 HE and Masson’s trichrome staining

Samples of the experimental teeth and their surrounding tissues in the root furcation area were excised and trimmed. The samples were then decalcified with 10% EDTA for 3 months. These samples were subsequently prepared for HE and Masson’s trichrome staining. HE staining was used to observe the formation of new alveolar bone, periodontal ligament, and cementum. On the other hand, Masson’s trichrome staining was used to assess new bone maturation and the formation of new collagen fibers. After 6 weeks, the main organs (the heart, liver, spleen, lungs, and kidneys) from each group were collected, fixed, dehydrated, and embedded in paraffin. The tissues were subsequently sectioned and stained with HE to evaluate whether the main organs were damaged.

### 2.9 RNA sequencing analysis

The periodontium was extracted from rats treated with BPCNs and PBS. RNA was isolated from the periodontium with TRIzol reagent. RNA sequencing was performed by BGI Genomics (China). Differentially expressed genes (DEGs) were analyzed via the edgeR analysis package in the R statistical program, with the criteria defined as an adjusted p value ≤0.05 and an absolute log2 (fold change) >1.5. Prism software (GraphPad) and RStudio were used to create heatmaps and volcano plots. Gene Ontology (GO) term enrichment analysis was performed for the top 200 deregulated DEGs. Gene set enrichment analysis (GSEA) was performed using GSEA software (https://www.gsea-msigdb.org/gsea/index.jsp).

### 2.10 Statistical analysis

All the data are presented as the means ± SEMs from at least three independent experiments. Comparisons between groups were performed using an unpaired two-tailed Student’s t-test or one-way analysis of variance (ANOVA) with Tukey’s *post hoc* test. A value of *p* < 0.05 was considered to indicate statistical significance. All the statistical analyses were carried out using Prism software (GraphPad).

## 3 Results

### 3.1 Preparation and characterization of BPCNs

**FIGURE 2:**
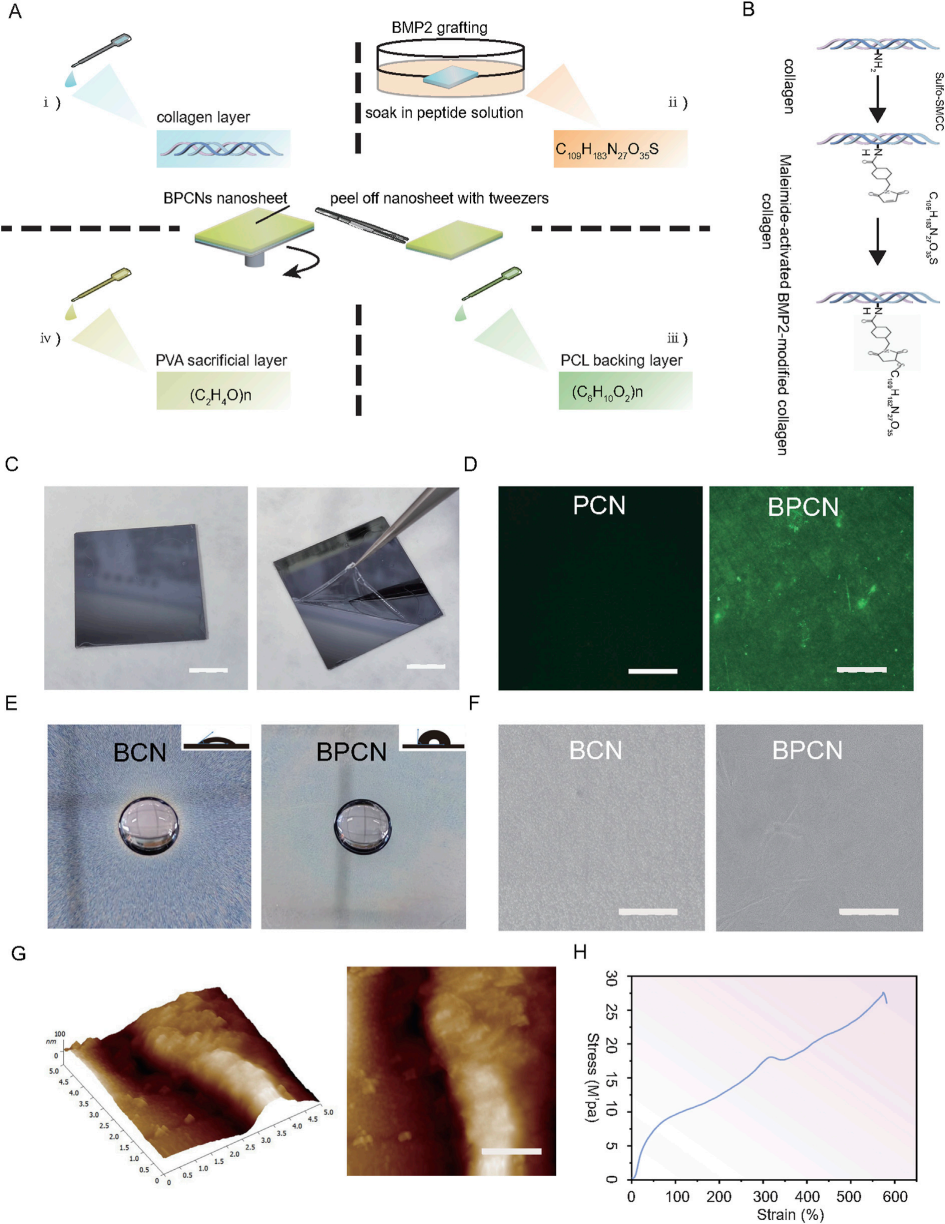
Preparation and characterization of the nanosheets. **(A)** BMP-modified PCL-collagen nanosheets (BPCNs) were prepared via the spin-coating technique. **(B)** C_109_H_183_N_27_O_35_S (KIPKASSVPTELSAISTLYLSGGC) was grafted onto the collagen layer via a thiol–maleimide reaction. **(C)** Peeling of the PVA-supported BPCNs from the silicon wafer. Scale bar = 1 cm. **(D)** CLSM images of the PCNs and BPCNs marked with FITC. Scale bar = 500 μm. **(E)** Water resistance and hydrophilicity of BCNs and BPCNs. Scale bar = 500 mm. **(F)** SEM images of the BCNs and BPCNs. Scale bar = 5 μm. **(G)** AFM images of the cross-sectional profile and the surface of the BPCNs. **(H)** Representative tensile strain-stress curve of BPCNs. Scale bar = 1 μm.

We first synthesized BPCNs using the spin coating technique (Figure 2A), which was chosen for its simplicity and flexibility. To graft the collagen layer with BMPs, the collagen layer was soaked in a polypeptide solution (Figure 2B). After grafting, we determined the concentration of the BMP2 peptide solution and calculated the amount and ratio grafted onto the BPCNs on the basis of the reaction between the sulfhydryl groups of C_109_H_183_N_27_O_35_S and DTNB (Ellmann’s reagent), which results in the formation of yellow substances. When 0.5 mg mL^−1^ BMP2 peptide solution (1.5 mg BMP2 dissolved in 3 mL EDTA solution) was used for grafting, the amount and ratio of KIPKASSVPTELSAISTLYLSGGC grafted onto the BPCNs were approximately 39.42 nmol cm^−2^ and 57%. To observe whether BMP2 peptide was grafted onto the nanosheets, C_109_H_183_N_27_O_35_S labeled with a FITC fluorescence group was utilized. Fluorescence microscopy revealed that the BPCNs exhibited strong fluorescence, indicating successful grafting of BMP2 peptide onto the nanosheets. In contrast, the PCNs without BMP2 peptide grafting did not exhibit fluorescence (Figure 2D). Finally, to ensure easy peeling of the nanosheets from the silicon wafer, a micron-thick PVA layer was introduced as a sacrificial layer on the BPCNs (Figure 2C).

Moreover, we examined the hydrophilicity and water resistance properties of the nanosheets, as the hydrophilicity of these nanosheets has a significant effect on cell adhesion and proliferation. After 1 hour of immersion, the water-soaked sections of both the BCNs and BPCNs remained insoluble, suggesting that both the PCL layer and the BMP2 peptide-modified collagen layer exhibited water resistance characteristics. Photographs captured to evaluate the water contact angles revealed that the collagen layer exhibited excellent hydrophilicity, whereas the PCL layer exhibited hydrophobicity (Figure 2E). After the PVA layer was washed away, we characterized the surface morphology of the BPCNs and BCNs by scanning electron microscopy and atomic force microscopy.

SEM observations revealed that the BPCN nanosheets are composed of multilayer structures, with distinct compact and porous layers. Additionally, the surfaces of the PCL layer and collagen layer appeared smooth (Figure 2F). Furthermore, AFM analysis provided insights into the cross-sectional profile and surface morphology of the BPCNs, revealing that they have a thickness of 83.49 nm ± 7.12 nm, which satisfied the required thickness for the nanosheets (Figure 2G).

We conducted tensile tests to measure the tensile properties of the nanosheets, considering that the nanosheets may suffer from stretching during GTR surgery. The tensile strain‒stress curve indicated that the BPCN nanosheets have great flexibility. The Young’s modulus of the BPCNs was approximately 27 MPa. These results suggest that the BPCNs are highly suitable for irregular defects (Figure 2H).

### 3.2 Cellular behavior and biocompatibility of BPCNs

**FIGURE 3:**
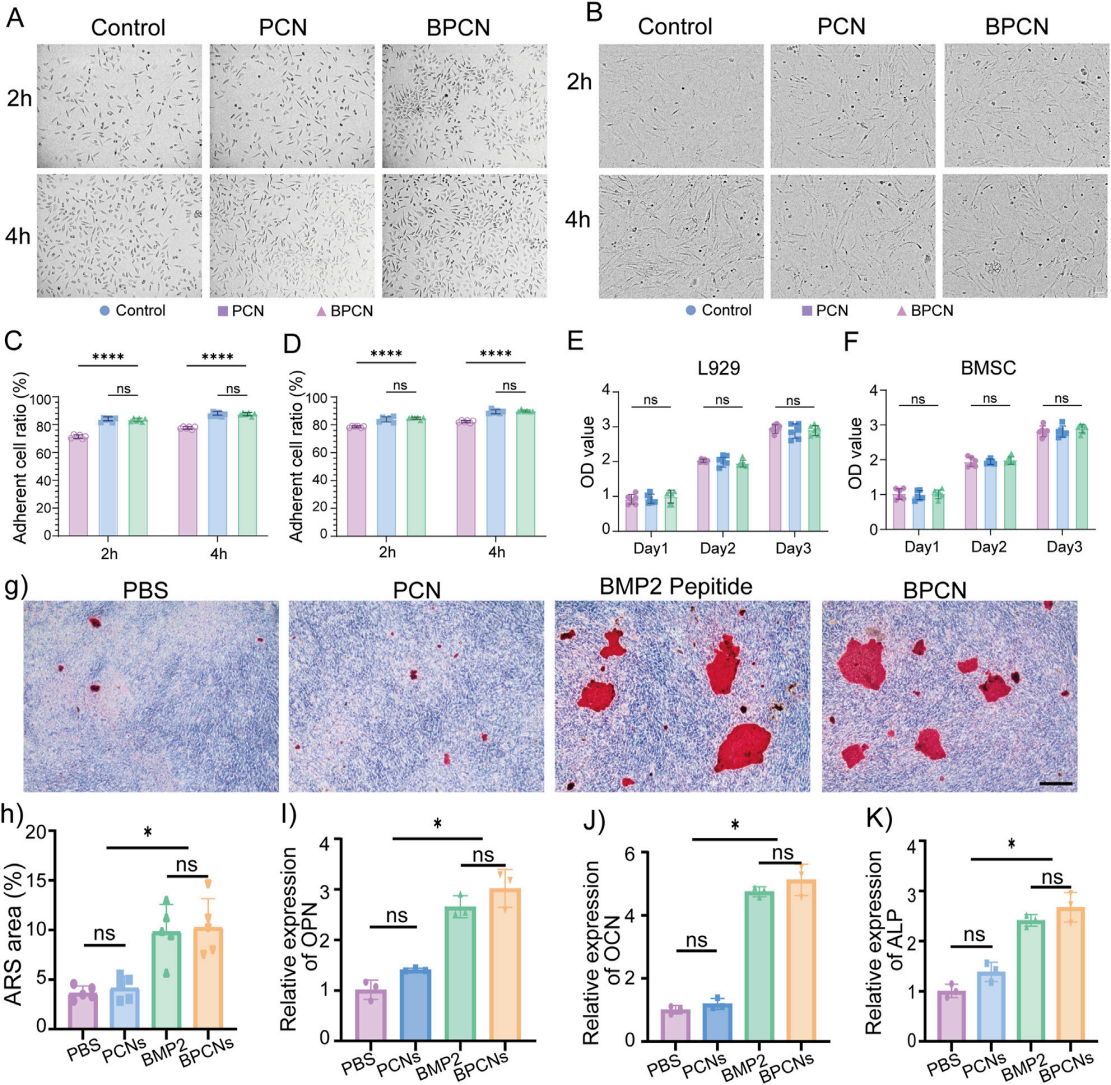
Cell adhesion properties, biocompatibility and osteogenic ability of BPCNs. **(A)** Images of L929 cell adhesion on various substrates. Scale bar = 50 μm. **(B)** Images of BMSC adhesion on various substrates. Scale bar = 50 μm. **(C)** The proportions of adherent L929 cells at 2 h and 4 h were calculated and are presented in a graph (****p < 0.0001). **(D)** The proportions of adherent BMSCs at 2 h and 4 h were calculated and are presented in a graph (****p < 0.0001). **(E)** The OD values obtained from the CCK8 assay of L929 cells on days 1, 2 and 3. **(F)** OD values obtained from the CCK8 assay of BMSCs on days 1, 2 and 3. Scale bar = 100 μm. **(G)** Images of ARS-stained areas on various substrates. Scale bar = 50 μm. **(H)** Proportions of ARS-stained areas on various substrates. **(I–K)** qRT‒PCR analysis of BMSC osteogenesis (OPN, OCN, ALP) (*p < 0.05).

A fundamental prerequisite for the utilization of biomaterials *in vivo* is excellent biocompatibility. Cellular behaviors such as viability, morphology, and adhesion were assessed by microscopy and the CCK8 assay. PCNs and BPCNs were used as the experimental groups to explore the cell adhesion ability of the collagen layer and BMP2 peptide-modified collagen layer. L929 cells and BMSCs were seeded onto plates containing a collagen layer. The cells were subjected to microscopy and photographed at 2 and 4 h, as shown in Figures 3A, B. Notably, as shown in Figures 3C, D, the numbers of adherent cells in the experimental groups were significantly greater than that in the PBS group, indicating that the collagen layers of both the PCNs and BPCNs accelerated the adhesion of L929 cells and BMSCs. Additionally, a CCK8 assay was conducted to evaluate cell proliferation and cytotoxicity over a period of 3 days. Compared with the PBS group, both the BPCN and PCN treatment groups exhibited no cytotoxicity (Figures 3E, F).

### 3.3 BPCNs increase the osteogenic ability of BMSCs

Calcium nodules are characteristic markers for osteogenesis within the bone structure. To assess osteogenesis, calcium nodules were observed in the Alizarin red S (ARS)-stained area on day 21. The results indicated that BMP2 peptide treatment and BPCN treatment led to the accumulation of calcium products in larger amounts, suggesting greater osteoinductive capacity (Figures 3G, H). Furthermore, the expression of osteogenesis-related genes, including OPN, ALP, and OCN, was examined to evaluate osteopromotive effects. Notably, as shown in Figures 3I–K, the expression of these osteogenesis-related genes was significantly upregulated in both the BMP2 peptide treatment group and the BPCN treatment group compared with that in the PBS group and PCN treatment group.

### 3.4 BPCNs show clear osteogenic capability *in vivo*

**FIGURE 4:**
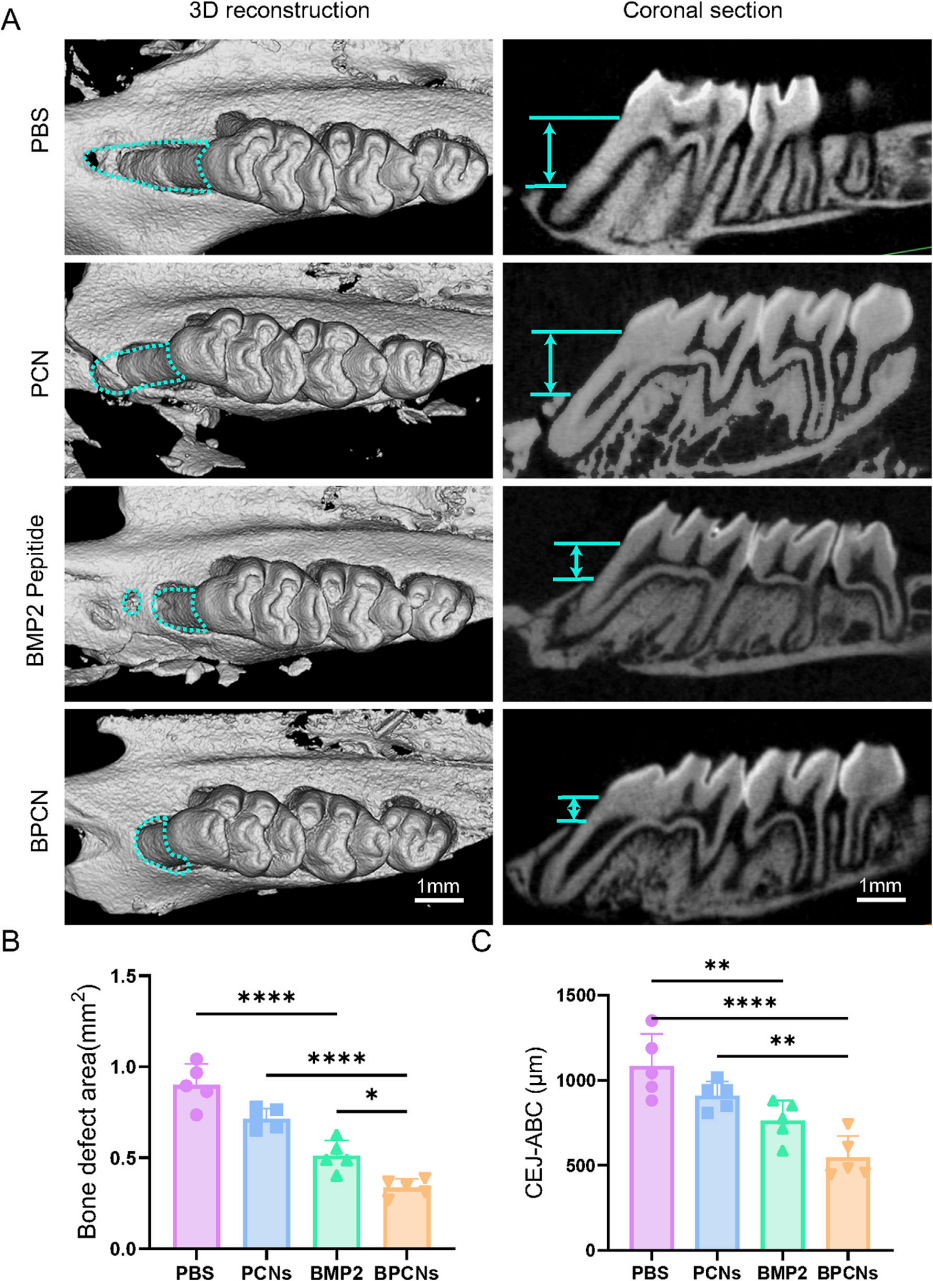
Alveolar bone repair in a rat periodontal defect model. **(A)** Micro-CT images of 3D reconstructions and buccopalatal sections of maxillary molars subjected to different treatments. The green lines represent the area of the bone defect and the distance between the ABC and the CEJ (scale bar = 1 mm). **(B)** Quantitative evaluation of the bone defect area. **(C)** Quantitative evaluation of the distance between the ABC and the CEJ. (*p < 0.05, **p < 0.01, ***p < 0.001, ****p < 0.0001).

The ability of BPCNs to guide the repair and regeneration of periodontal defects was evaluated in a rat model. A periodontal defect was surgically created by removing a segment of the alveolar bone to mimic a clinical periodontal defect. As shown in Figure 4A, gross observation after 6 weeks revealed that the rats in the PBS group exhibited the least amount of new bone formation. The defect remained obvious, with exposed root surfaces, indicating limited spontaneous healing capabilities. On the other hand, compared with the PBS group, the PCN group presented slightly more new bone formation. This increase in bone formation suggested that the PCL material and collagen material might have some osteogenic potential, although the effect was still relatively limited. In contrast, the BMP2 peptide treatment significantly promoted osteogenesis at the defect site, as evidenced by substantial new bone formation within the periodontal defect. BMP2, a well-known osteogenic factor, strongly promoted osteogenesis at the defect site. Interestingly, combined treatment with PCNs and BMP2 peptide resulted in an even more pronounced osteogenic effect. The newly formed bone in the BPCN treatment group almost filled the defect, resulting in nearly complete restoration of the periodontal structure. As shown in Figures 4B, C, both the BMP2 peptide treatment group and the BPCN treatment group presented obvious decreases in the bone defect area and the length of the CEJ-ABC. Moreover, in the BPCN group, the bone defect area and the length of the CEJ-ABC were reduced the most among all the groups. These findings suggest that the combination of PCNs and BMP2 peptide could synergistically enhance the repair and regeneration of periodontal defects.

### 3.5 BPCNs promote tissue regeneration *in vivo*

**FIGURE 5:**
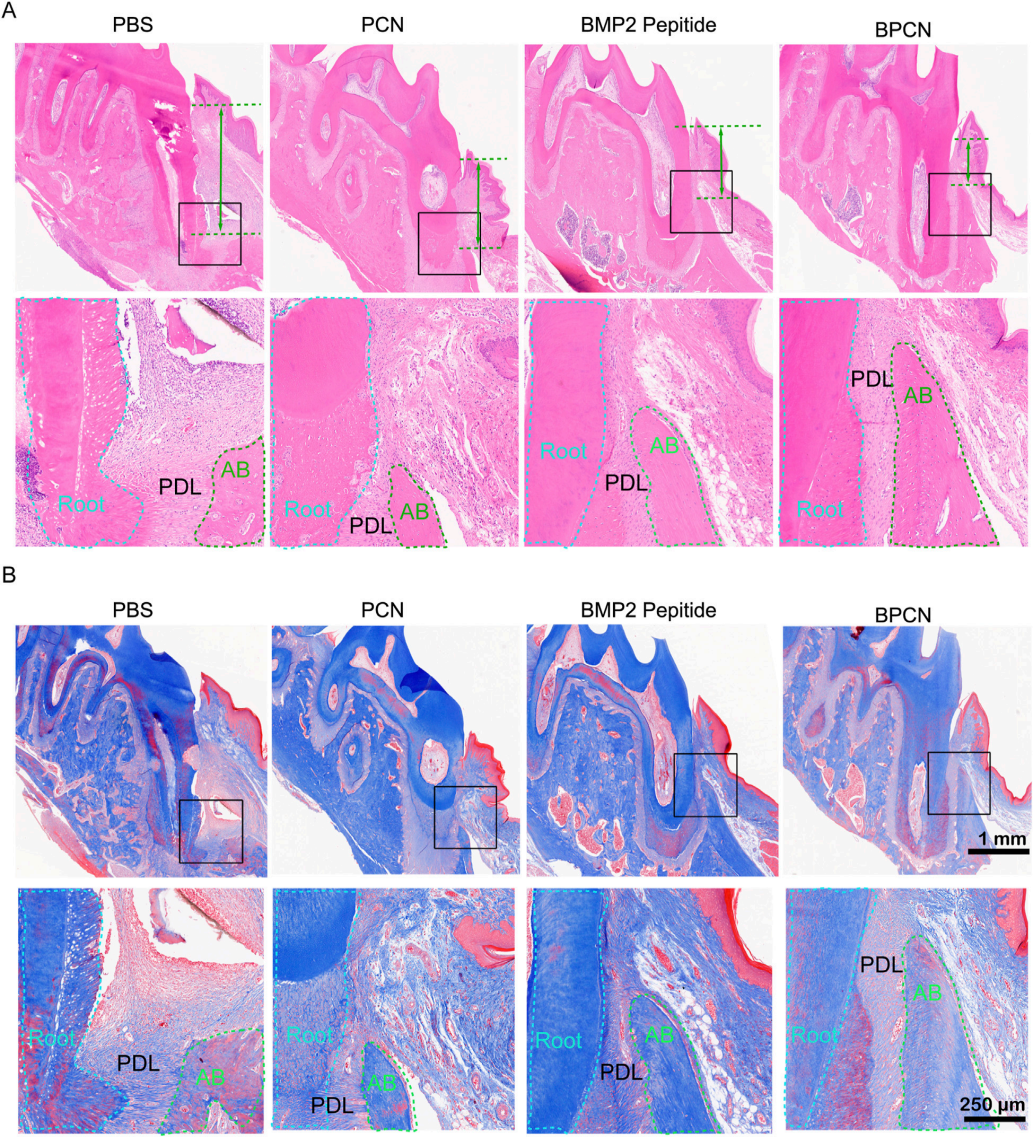
Tissue repair in a rat periodontal defect model. **(A)** H&E staining of periodontal tissue. **(B)** Masson staining of periodontal tissue. The green lines represent the distance between the ABC and the CEJ.

**FIGURE 6:**
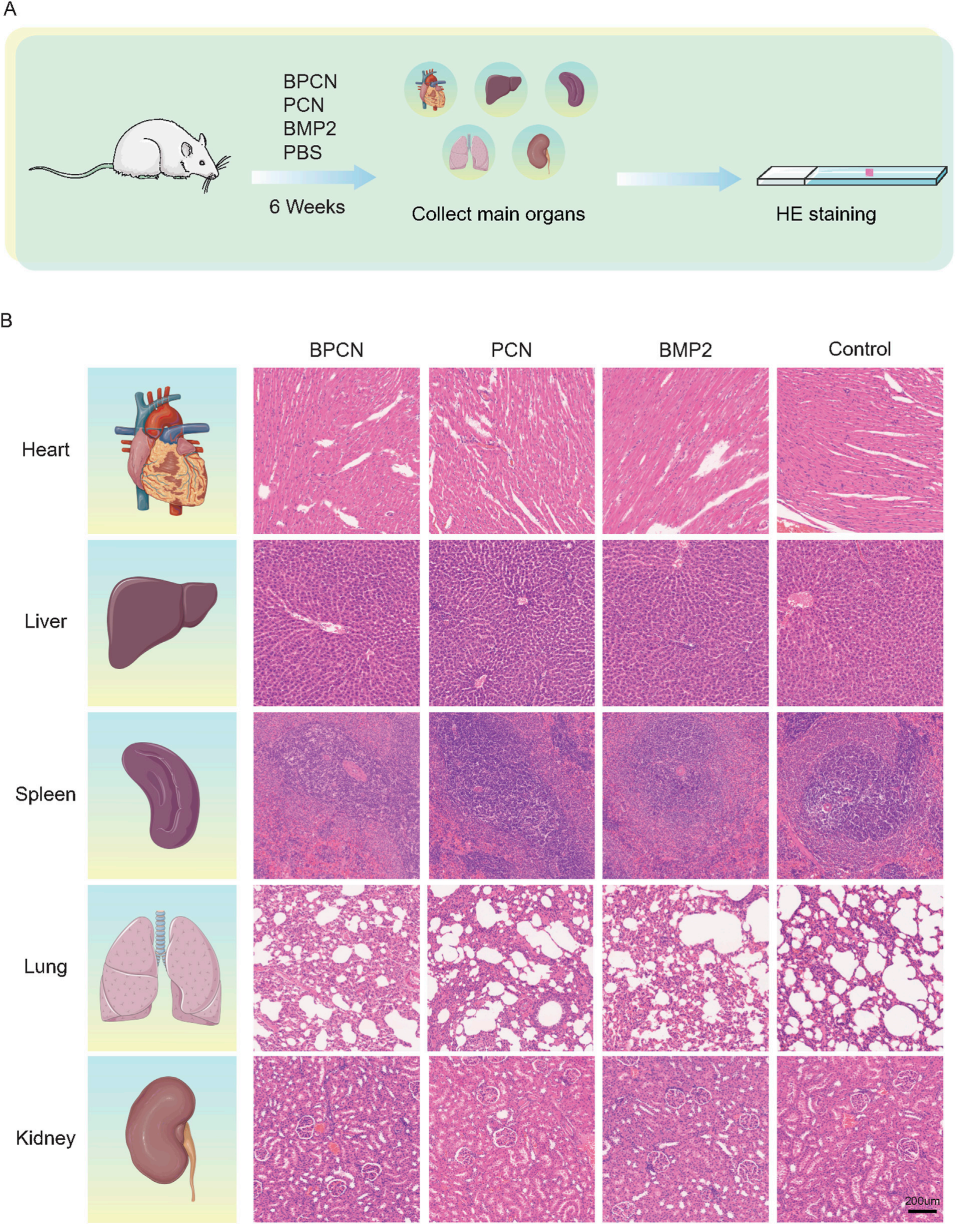
BPCNs exhibit excellent biocompatibility and biosafety in vivo. **(A)** Technical roadmap for visceral sampling in rats. **(B)** H&E staining of rat major organs (the heart, liver, spleen, lungs, and kidneys) after nanosheet treatment for 6 weeks. Scalebar = 200 μm.

To further investigate and evaluate guided periodontal tissue regeneration by BPCNs, the rats were sacrificed and subjected to histological staining. As shown in Figures 5A, E, H staining revealed that the PBS group exhibited dense connective tissue but lacked new bone formation. This observation suggested that without any additional treatment, the periodontal tissue regeneration of the rats was limited. In contrast, the other three groups displayed varying degrees of new bone formation, with a small amount of new bone observed in the PCN group. This finding was encouraging, indicating that PCNs have some regenerative potential, but the effect was relatively small. Moreover, the BMP-2 peptide group presented more new bone formation. The periodontal defects in the BPCN group were mostly filled by regenerated alveolar bone. As shown in Figure 5B, Masson staining was carried out to evaluate the collagenous matrix of the regenerated alveolar bone, as high-quality newly synthesized collagenous matrix is an important indicator for regenerated alveolar bone tissue and exhibits dark blue staining. Limited collagenous matrix staining was observed in the PBS group, whereas varying intensities of dark blue collagenous matrix staining were observed in the other three groups. Notably, the intensity of the dark blue staining closely corresponded to the amount of regenerated bone. H&E staining of the major organs (Figure 6) indicated that the BMP2 peptide, BPCN and PCN treatments did not significantly affect the major organs of SD rats.

### 3.6 BPCNs upregulate the expression of components of pathways related to tissue regeneration but downregulate the expression of components of proinflammatory pathways

**FIGURE 7:**
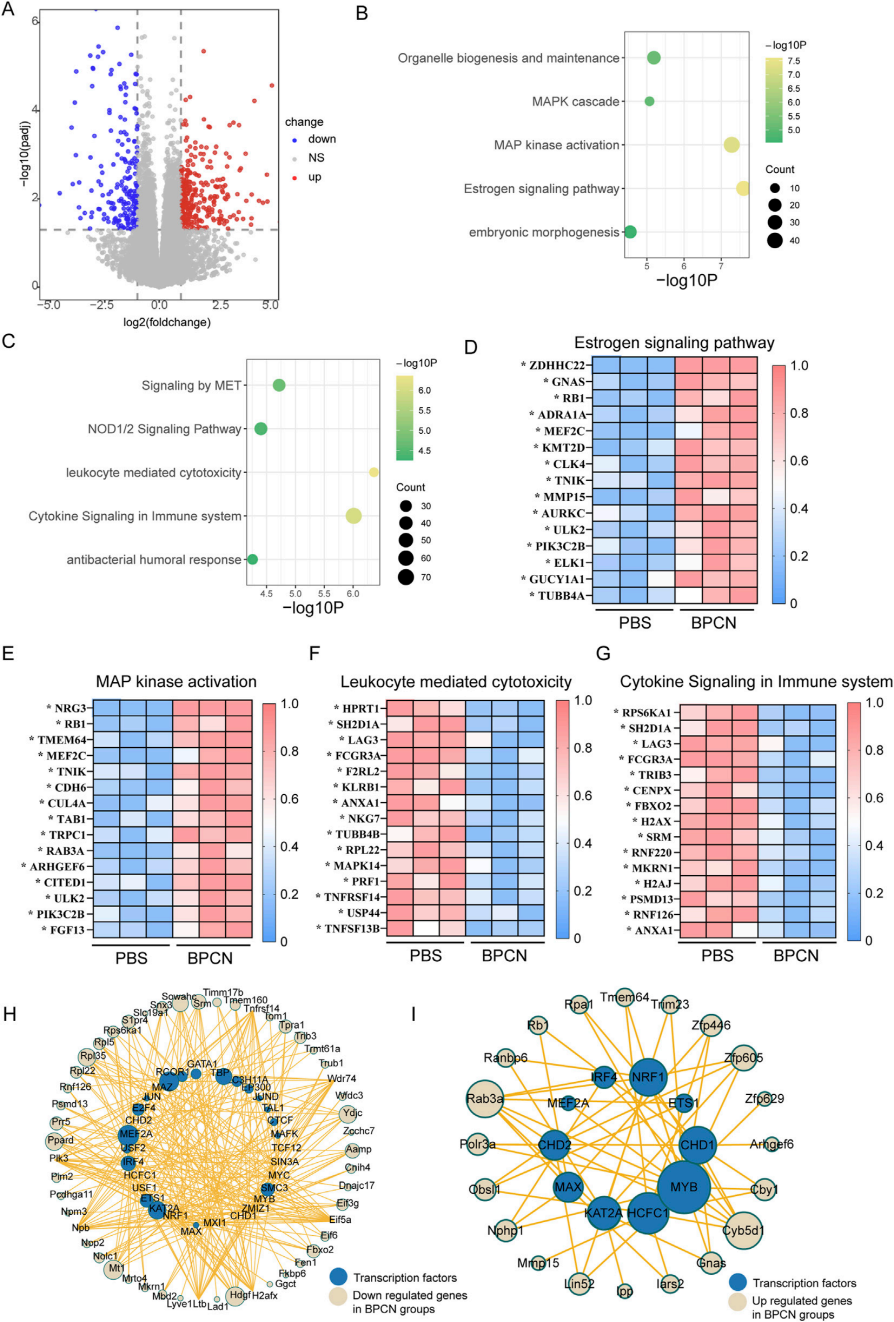
BPCNs upregulate components of pathways related to tissue regeneration but downregulate components of proinflammatory pathways. **(A)** Volcano plots showing DEGs. **(B)** Gene Ontology (GO) functional analysis of upregulated DEGs in the periodontium of the BPCN-treated group compared with the PBS-treated group. **(C)** GO functional analysis of downregulated DEGs in the periodontium of the BPCN-treated group compared with the PBS-treated group. **(D–G)** Heatmaps of DEGs from the selected pathways in the periodontium of the BPCN-treated group compared with the PBS-treated group. *p < 0.05. **(H, I)** Analysis of transcription factors related to inflammatory genes.

We conducted RNA sequencing analysis of the periodontium of rats with periodontitis that were treated with BPCNs or PBS to elucidate the mechanisms underlying the therapeutic effects of BPCNs in periodontitis model rats. As shown in Figure 7A, in the periodontium of BPCN-treated and PBS-treated rats with periodontitis, we identified differentially expressed genes (DEGs) (adjusted p value ≤0.05 and absolute log2 (fold change) >1.5). To gain further insight, we conducted a GO term enrichment analysis of the DEGs on the basis of their involvement in biological processes. Compared with those in PBS-treated rats with periodontitis, the upregulated DEGs in the periodontium of BPCN-treated mice were enriched predominantly in terms such as the estrogen signaling pathway and MAP kinase activation (Figure 7B), whereas the downregulated DEGs were enriched in terms such as the leukocyte-mediated cytotoxicity pathway and cytokine signaling in the immune system (Figure 7C). Gene set enrichment analysis (GSEA) further confirmed activation of the identified pathways (Supplementary Figure S1).

Heatmaps of the DEGs within these enriched GO pathways revealed that DEGs associated with the estrogen signaling pathway and MAP kinase activation were upregulated in BPCN-treated rats with periodontitis compared to PBS-treated rats with periodontitis, whereas leukocyte-mediated cytotoxicity and cytokine signaling in the immune system were downregulated (Figures 7D–G). We conducted transcription factor analysis on the upregulated and downregulated genes and found that the downregulated genes correspond to transcription factors MAZ, KAT2A, and NFRF1, which are associated with inflammation, indicating that treatment can downregulate inflammation. At the same time, upregulated genes correspond to transcription factors MYB, HCFC1, and MAX, which are associated with tissue regeneration. In summary, BPCNs can promote periodontal defect repair by downregulating inflammation and promoting tissue regeneration (Figures 7H, I). Taken together, the findings from the GO term enrichment analysis suggest that BPCN treatment suppresses the immune and inflammatory response in rats with periodontitis.

## 4 Discussion

Barrier membranes are critical in guided tissue regeneration (GTR) surgery (Sanz et al., 2020), as they prevent soft tissue invasion and allow time for tissue repair (Donos et al., 2023; Francisco et al., 2019). Our study demonstrated that BPCNs not only enhance cell adhesion and bone regeneration *in vitro* but also significantly improve periodontal tissue regeneration *in vivo*. RNA sequencing confirmed that BPCNs activate tissue regeneration pathways and reduce inflammation, making them promising for GTR surgery due to their ease of use and bone-inducing properties.

We employed nanomaterials and spin coating technology to fabricate collagen nanosheets specifically aimed at periodontal regeneration. While our previous work demonstrated that nanotechnology enabled the creation of thinner, more adhesive barrier membranes suitable for soft tissue defects (Fujie et al., 2009; Fu et al., 2023; Zhang Y. et al., 2024), it remains uncertain whether these nanosheets can fully address periodontal regeneration. Spin coating was selected for its simplicity, precision, and efficiency, facilitating rapid, large-scale production at a low cost (Xuan et al., 2020). Collagen, known for its excellent biocompatibility, promotes cell adhesion and proliferation, and can also serve as a carrier for bioactive peptides (Naomi et al., 2021; Sun et al., 2022; Xie et al., 2018; Cooperman and Michaeli, 1984; Calciolari et al., 2018; Niwa et al., 2012; Stoecklin-Wasmer et al., 2013). To enhance mechanical strength and performance, we integrated PCL as a backing layer, a material recognized for its biocompatibility and durability (Dwivedi et al., 2020; Hedvicakova et al., 2023). Our research confirmed that both PCL and collagen were non-toxic in SD rats, and their combination enhanced the overall functionality of the barrier membrane (Bezwada et al., 1995). The hydrophobicity of the PCL layer also supports controlled drug release, improves membrane stability within periodontal defects, and ensures an optimal degradation rate, making this combination promising for clinical applications (Dwivedi et al., 2020; Engelberg and Kohn, 1991; Gumusderelioglu et al., 2019).

Current clinical barrier membranes often lack bone-guiding capabilities, limiting their effectiveness in periodontal regeneration (Chen et al., 2021; Zhang Q. et al., 2024; Zhang et al., 2023). To address this, we aimed to enhance this function by incorporating BMP2 peptide, a potent osteogenic factor. The BMP2 peptide was chemically stabilized on the collagen layer, allowing for uniform distribution and prolonged bioactivity, which significantly improved osteogenic differentiation in BMSCs (Kim et al., 2013; Chen et al., 2021; Kim et al., 2018; Howard et al., 2022). In our study, BPCNs significantly accelerated bone regeneration in a rat model of periodontal defects. Micro-CT analysis confirmed that BPCNs markedly enhanced alveolar bone healing compared to control groups. Additionally, Masson’s trichrome staining revealed increased and organized collagen fiber regeneration, suggesting that BPCNs not only promoted bone healing but also supported soft tissue repair, accelerating the overall healing process. These findings demonstrate the significant therapeutic potential of BPCNs in treating periodontal defects. Their ability to promote both bone regeneration and soft tissue healing makes them a promising candidate for clinical applications in periodontal regeneration therapies.

We identified the mechanism by which BPCNs promote periodontal defect healing. In the BPCN treatment group, the estrogen and MAP kinase pathways were significantly upregulated. Estrogen inhibits osteoclast formation and promotes their apoptosis, reducing osteoclast numbers and bone resorption, while also stimulating osteoblast proliferation and differentiation, enhancing bone formation (Almeida et al., 2017). Additionally, MAP kinase pathway activation is crucial for osteoblast differentiation (Almeida et al., 2017; Xiao et al., 2002). Inflammation, particularly when excessive and prolonged, impairs periodontal tissue regeneration (Gruber, 2019). Our results showed that factors associated with leukocyte-mediated cytotoxicity and cytokine signaling were significantly downregulated in the BPCN group, suggesting that BPCNs reduce inflammation during tissue regeneration, thus creating a more favorable environment for healing.

According to the S3-level clinical guidelines for periodontitis treatment issued by the European Federation of Periodontology, the use of barrier membranes in regenerative therapy is strongly recommended, with the option to either incorporate or omit bone grafts (Sanz et al., 2020; Nibali et al., 2020). BPCNs offer an environment conducive to bone cell growth and attachment. They optimize the local microenvironment by releasing bone morphogenetic protein-2 (BMP-2) peptide, directly promoting bone cell differentiation and bone formation, and facilitating natural repair of bone tissue. In our study, a rat model of periodontal tissue defects with relatively small defect volumes was established, and good regenerative outcomes were obtained without the use of bone graft materials. Therefore, the use of periodontal regeneration surgery without bone graft materials is feasible in some cases. Yet, further preclinical studies and clinical trials are necessary to verify their ability to achieve better regenerative effects in various types of defects and complex cases.

In conclusion, our study successfully utilized spin coating to fabricate BMP2 peptide-incorporated BPCNs, demonstrating their effectiveness in promoting periodontal tissue regeneration. These findings support the potential of PCL-collagen nanosheets as practical, bone-regenerative materials for GTR, characterized by their ease of use and ability to enhance tissue repair.

## Data Availability

The raw data supporting the conclusions of this article will be made available by the authors, without undue reservation.

## Abbreviations

PCL, Polycaprolactone; BPCNs, BMP2 peptide-modified collagen nanosheets; BMSCs, bone marrow stem cells; GTR, guided tissue regeneration; PVA, polyvinyl alcohol; EDC, 1-ethyl-3-(3-dimethylaminopropyl) carbodiimide; NHS, N-hydroxy succinimide; EDTA, ethylenediaminetetraacetic acid; CNs, collagen nanosheets; BCNs, BMP2 peptide-modified collagen nanosheets; FITC, fluorescein isothiocyanate; SEM, scanning electron microscopy; AFM, atomic force microscopy; PCNs, PCL-collagen nanosheets; TCPS, tissue culture-treated polystyrene; OPN, osteopontin; OCN, osteocalcin; ALP, alkaline phosphatase; qRT‒PCR, quantitative real-time polymerase chain reaction; CEJ‒ABC, cementoenamel junction and the alveolar bone crest; DEGs, differentially expressed genes; GO, Gene Ontology; GSEA, gene set enrichment analysis, ARS, alizarin red S.
