# Supplementary material for: BMP2 peptide-modified polycaprolactone-collagen nanosheets for periodontal tissue regeneration
Source: Front Bioeng Biotechnol. 2025 Mar 5;13:1523735. doi: 10.3389/fbioe.2025.1523735 (PMC11919852; doi:10.3389/fbioe.2025.1523735)
Supplement: Supplementary file 1 [file DataSheet1.pdf]

Supplementary Material

1 Supplementary Figures

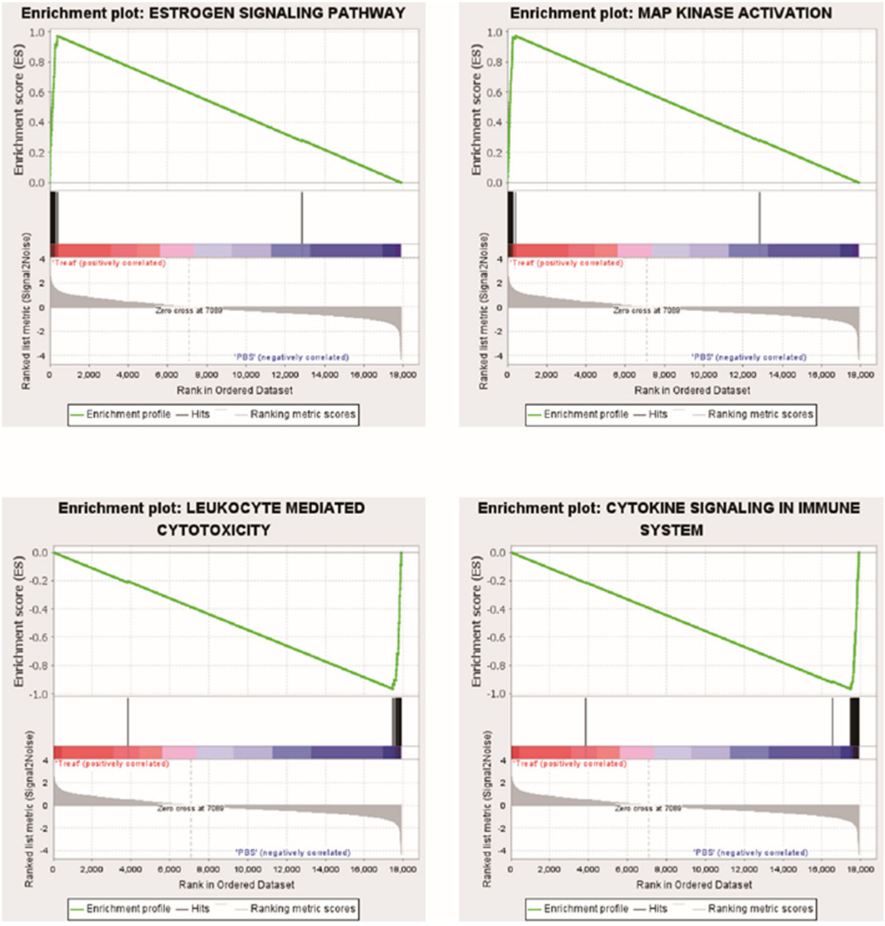

Fig. S1 Gene set enrichment analysis (GSEA) of the signature genes in the periodontium of the BPCN-treated group compared with the PBS-treated group.

2 Supplementary Tables

Supplementary Table 1 Primer sequences used for qRT–PCR

Gene	Forward primer (5’-3’)	Reverse primer (5’-3’)
OCN	TATGGCACCACCGTTTAGGG	CTGTGCCGTCCATACTTTCG

OPN	CTGCCAGCACACAAGCAGAC	TCTGTGGCATCGGGATACTG
ALP	TCCCAAAGGCTTCTTCTTGC	ATGGCCTCATCCATCTCCAC
GAPDH	TTCAACGGCACAGTCAAG	TACTCAGCACCAGCATCA
